# Analysis of the incidence and influencing factors of abdominal distension in postoperative lung cancer patients in ICU based on real-world data: a retrospective cohort study

**DOI:** 10.1186/s12893-024-02317-2

**Published:** 2024-01-18

**Authors:** Yan Liu, Tingting Tang, Chunyan Wang, Chunmei Wang, Daxing Zhu

**Affiliations:** 1 Department of Critical Care Medicine, West China Hospital, Sichuan University, Chengdu, Sichuan, China; 2 Department of Thoracic Surgery, West China Hospital, Sichuan University, Chengdu, Sichuan, China; 3 Lung Cancer Center, West China Hospital, Sichuan University, Chengdu, Sichuan, China

**Keywords:** Lung cancer, Surgical resection, Abdominal distension

## Abstract

**Background:**

Abdominal distension is a relatively common complication in postoperative lung cancer patients, which affects patients’ early postoperative recovery to varying degrees. However, the current status of the incidence of abdominal distension in postoperative lung cancer patients and the affecting factors are not well understood. This study aims at exploring the incidence of abdominal distension in postoperative lung cancer patients in ICU based on real-world data and analyzing its influencing factors.

**Methods:**

A retrospective cohort study was conducted, encompassing patients who underwent lung cancer resections in the Lung Cancer Center of West China Hospital of Sichuan University from April 2020 to April 2021. Nevertheless, patients younger than 18 years and those whose information was limited in medical records were excluded. All data were obtained from the hospital HIS system. In this study, the influencing factors of abdominal distension were analyzed by univariate analysis and multiple logistic regression methods.

**Results:**

A total of 1317 patients met eligibility criteria, and were divided into the abdominal distended group and the non-distended group according to whether abdominal distension occurred after surgery. Abdominal distension occurred in a total of 182 cases(13.8%). The results of the univariate analysis showed that, compared with the non-distended group, the abdominal distended group had these features as follows: more women (*P* = 0.021), older (*P* = 0.000), lower BMI (*P* = 0.000), longer operation duration (*P* = 0.031), more patients with open thoracotomy (*P* = 0.000), more patients with pneumonectomy (*p* = 0.002), more patients with neoadjuvant chemotherapy (*P* = 0.000), more days of hospitalization on average (*P* = 0.000), and higher costs of hospitalization on average (*P* = 0.032). Multifactor logistic regression analysis showed that sex (*OR* = 0.526; 95% *CI* = 0.378 ~0.731), age (*OR* = 1.154; 95%*CI* = 1.022 ~1.304) and surgical approach (*OR* = 4.010; 95%*CI* = 2.781 ~5.781) were independent influencing factors for the occurrence of abdominal distension in patients after lung cancer surgery in ICU.

**Conclusions:**

The incidence of abdominal distension was high in postoperative lung cancer patients in ICU, and female, older and patients with open thoracotomy were more likely to experience abdominal distension.

**Trial registration:**

The study was approved by the Chinese Clinical Trials Registry (registration number was ChiCTR2200061370).

## Background

Lung cancer is the leading cause of cancer deaths world-wide [1]. In China, lung cancer has become the fastest growing malignancy in terms of incidence rate and mortality in the last 30 years [2].

Available treatments for lung cancer include surgery, chemotherapy, immunotherapy, and radiotherapy [3]. Surgery is the primary treatment for early-stage lung cancer patients. And surgery also showed efficacy in advanced stage disease after induction therapy in the context of multidisciplinary treatment models [4–8].Studies showed that a series of complications may occur after lung cancer surgery, including pulmonary infection, respiratory failure, cardiac arrhythmia and acute heart failure [9, 10].

In clinical work, we found that surgical trauma, anesthetic mechanicals, and mechanical ventilation predispose patients to abdominal distension after lung cancer surgery, which is defined as a measurable increase in abdominal girth, mainly manifested by gas retention, increased intra-abdominal pressure, and a feeling of fullness [11], which affects patients’ early postoperative recovery. However, compared with other serious complications, postoperative abdominal distension after lung cancer is often easily overlooked, and there are few studies on abdominal distension in postoperative patients with lung cancer, and its course and the influencing factors are not very clear.

Hospital Information System (HIS system) is mainly based on patient information, financial information and material information to provide timely, comprehensive and accurate data sources for hospital personnel in all departments, using the method of collection, storage, transmission, statistics, analysis, comprehensive query, report output and information sharing of information. The objective of this study is to report demographic and clinical characteristics of postoperative patients with lung cancer during 2020–2021 based on real-world data from the HIS system. The focus is to report the incidence of abdominal distension in postoperative lung cancer patients in ICU and to analyze its influencing factors.

## Methods

### Patients

Included in this study were patients who underwent surgical resections in lung cancer center of West China Hospital of Sichuan University between April 1, 2020 and April 30, 2021.

The inclusion criteria were (1) patients who were pathologically confirmed lung cancer and require surgery; (2) patients who were 18 years and older; (3) patients whose complete case information was available; (4) patients who were transferred to ICU for treatment after lung cancer surgery.

The exclusion criteria were (1) patients whose symptoms were combined with gastrointestinal diseases and other serious diseases such as liver cirrhosis, (2) patients whose data was not completed.

### Research methods

#### Research design

The study was a retrospective cohort study. The retrospective data was obtained from the HIS system. The study was approved by the hospital ethics committee with the approval number 2022 review (No. 1046) and the registration number ChiCTR2200061370 of the Chinese Clinical Trials Registry. The informed consent form was waived with the approval of the Bioethics Review Committee of West China Hospital, Sichuan University.

### Data collection

Two investigators look up medical records through the HIS system to collect demographic and clinically relevant information of the patients, including sex, age, BMI, surgical approach, basic medical history, chemotherapy, postoperative pain level, the use of the analgesic pump after operation, the time of the intubation and extubation of the trachea, blood potassium, blood calcium, and the length of ICU stay, days of hospitalization on average, and costs of hospitalization on average.

Judgment criteria for the degree of abdominal distension [12]:

Grade 0: no abdominal distension;

Grade 1: mild abdominal distension, which does not affect sleep and rest or eating;

Grade 2: moderate abdominal distension, affecting sleep and rest and delaying eating;

Grade 3: severe abdominal distension, which does not allow normal sleep and rest and requires gastrointestinal decompression.

If necessary, combine with the X-ray/CT report to comprehensively determine whether the patient has abdominal distension and the degree of abdominal distension.

### Statistical analysis

Patients were grouped according to whether abdominal distention occurred by combining their symptoms and abdominal X-ray/CT reports, and SPSS 26.0 software was used for data processing and statistical analysis.

The above 17 risk factors were analyzed by univariate analysis (sex, age, BMI, TNM stage, operation duration, sedation and the amount of curarization time, postoperative extubation time, surgical approach, type of surgical resection, basic medical history, neoadjuvant chemotherapy, postoperative pain level, postoperative opioid analgesic use, the time to start oral intake, whether there is gas or stool output in ICU period, blood potassium, blood calcium). Significant differences in univariate analysis (sex, age, BMI, operation duration, surgical approach, type of surgical resection, neoadjuvant chemotherapy) were subjected to multivariate logistic regression analysis. Univariate analysis of the three outcome indicators (length of stay in the intensive care unit, mean number of days in the hospital, and mean hospital costs). The univariate analysis of common postoperative complications of lung cancer (lung infection, cardiac arrhythmia and hemorrhage) was performed, to further explore the influencing factors on the total number of days of hospitalization and the costs of hospitalization of the patients. Statistical significance of the results was indicated as *P* < 0.05.

## Results

The findings showed that among 1317 postoperative patients with lung cancer in ICU who met the criteria of adbdominal distension, 182 cases(13.8%) had different degrees of abdominal distension.

Compared with the non-distended group, the abdominal distended group were more women (*P* < 0.05), older (*P* < 0.01), and lower BMI (*P* < 0.01), longer operation duration (P＜0.05). A higher proportion of open thoracotomy (21.44% incidence of distension) patients experienced abdominal distension compared with thoracoscopic surgery (6.94% incidence of distension) (*P* < 0.01). The icidence of abdominal distention was higher in patients with pneumonectomy (23.08% incidence of distension) compared with other types of surgical resection (P＜0.01). The incidence of abdominal distention was higher in patients with neoadjuvant chemotherapy (25%) compared with those who did not (12.62%) (*P* < 0.01), as detailed in Table 1.

It is worth noting that the use of perioperative anesthesia and analgesics has been standardized for all lung cancer patients in our hospital, analgesic pumps were used in all postoperative patients, and additional opioid analgesic were used in some patients with severe postoperative pain.

Firstly, the indicators with statistical significance in the results of univariate analysis were selected as independent variables, including sex (man 1, and woman 0), age (18∼20 as 1, 21∼30 as 2, 31∼40 as 3, 41∼50 as 4, 51∼60 as 5, 61∼70 as 6, and 71∼80 as 7), BMI (raw value), operation duration(raw value), surgery approach (open thoractomy 1, and VATS 0), type of surgical resection wedge resection 1, segmentectomy 2, lobectomy 3, pneumonectomy 4, and others and neoadjuvant chemotherapy or not (no 0, and yes 1). Whether abdominal distension occurred after surgery was used as the dependent variable, and a multifactorial logistic regression analysis was performed. The results showed that gender, age and surgery apporoch were independent risk factors for the occurrence of abdominal distension in postoperative patients with lung cancer, as detailed in Table 2.

Compared with the non-distended group, patients in the abdominal distended group had more days of hospitalization on average(*P* < 0.01) and higher costs of hospitalization on average (*P* < 0.05), but there was no statistical difference in the length of ICU admission (*P* > 0.05), as detailed in Table 3.

Since a series of complications may be combined after lung cancer surgery, which perhaps leading to prolonged the days of hospitalization and the costs of hospitalization, thus we further analyzed the incidence of common complications after lung cancer surgery and their impact on the days of hospitalization and the costs of hospitalization of the patients.

The results showed that patients who developed abdominal distension group after lung cancer surgery had a higher incidence of complications such as lung infection, cardiac arrhythmia, and hemorrhage than Non-distended group, but none of the differences were statistically significant (*P* > 0.05), as detailed in Table 4.

Patients with lung infection had higher hospitalization days and hospitalization costs (*P* < 0.05). Patients with cardiac arrhythmia and hemorrhage had higher hospitalization days and hospitalization costs compared to patients in the normal group, however, the differences were not statistically significant (*P* > 0.05). Pneumonectomy patients had higher hospitalization days and hospitalization costs (*P* < 0.05), as detailed in Table 5.

**Table 1:** Comparison of risk factors between abdominal distension and non-distended groups (*n* = 1317)

Group	Abdominal distension group (*n* = 182)	Non-distended group (*n* = 1135)	*P*
Women (M/W), *n*(*%*)	107(58.79)	563(49.60)	0.021*
Age (yr.), *M* ( *Q*_1_, *Q*_3_)	55.5(48,63.25)	57(50,66)	0.000*
Age (yr.), *n*(*%*)			0.000*
18∼20	5(2.75)	9(0.79)	
21∼30	5(2.75)	21(1.85)	
31∼40	11(6.04)	76(6.70)	
41∼50	41(22.53)	185(16.30)	
51∼60	66(36.26)	359(31.63)	
61∼70	44(24.18)	350(30.84)	
71∼80	10(5.49)	135(11.89)	
BMI (kg/m^2^), *M* ( *Q*_1_, *Q*_3_)	23.23(21.31,24.98)	23.23(21.31,25.25)	0.000*
TNM stage, *n*(*%*)			0.077
I	118(64.84)	794(69.95)	
II	25(13.74)	176(15.51)	
III	27(14.83)	101(8.90)	
IV	12(6.59)	64(5.64)	
Operation duration (h), ($\overline x \pm s$)	1:50 ± 1:02	1:41 ± 0:55	0.031*
Sedation and the amount of curarization time (h), ($\overline x \pm s$)	4:17 ± 1:09	4:15 ± 1:09	0.793
Postoperative extubation time (hours), ($\overline x \pm s$)	6:11 ± 3:15	6:04 ± 3:03	0.595
Surgical approach, *n*(*%*)			0.000*
Open thoracotomy	134(73.63)	491(43.26)	
VATS	48(26.37)	644(56.74)	
Type of surgical resection, *n*(*%*)			0.002*
Wedge resection	16(8.79)	139(12.25)	
Segmentectomy	60(32.97)	269(23.70)	
Lobectomy	82(45.05)	642(56.56)	
Pneumonectomy	3(1.65)	10(0.88)	
Others	21(11.54)	75(6.61)	
Basic diseases history, *n*(*%*)			0.287
no	52(28.57)	282(24.85)	
yes	130(71.43)	853(75.15)	
Neoadjuvant Chemotherapy, *n*(*%*)			0.000*
no	150(82.42)	1039(91.54)	
yes	32(17.58)	96(8.46)	
Postoperative pain level, *n*(*%*)			0.359
mild	171(93.96)	1084(95.51)	
moderate	11(6.04)	51(4.49)	
Postoperative opioid analgesic use, n(%)			0.131
no	92(50.55)	508(44.76)	
yes	90(49.45)	627(55.24)	
The time to start oral intake, (h) ($\overline x \pm s$)	13:16 ± 3:10	12:50 ± 3:24	0.087
Whether there is gas or stool output in ICU period, n(%)			0.743
no	167 (91.76)	1033(91.01)	
yes	15(8.24)	102(8.99)	
Blood potassium (mmol/L), *n*(*%*)			0.516
low	60(32.97)	422(37.18)	
normal	121(66.48)	709 (62.47)	
high	1(0.55)	4(0.35)	
Blood calcium (mmol/L), *n*(*%*)			0.411
low	29(15.93)	155(13.66)	
normal	153(84.07)	980(86.34)	

BMI?Body mass index; Normal range of blood potassium?3.5∼5.5mmol/L; Normal range of blood calcium?2.25∼2.75mmol/L

* Statistically significant

**Table 2:** Logistic regression analysis of risk factors for abdominal distension

Factors	B	SE	Walds	***P***	OR	95%CI
Sex (M / F)	-0.643	0.168	14.641	0.000*	0.526	0.378∼0.731
Age (yr.)	0.144	0.062	5.314	0.021*	1.154	1.022∼1.304
BMI (kg/m^2^)	0.007	0.026	0.064	0.801	1.007	0.957∼1.059
operation duration (h)	0.000	0.000	1.537	0.215	1.000	1.000∼1.000
Surgical approach (open thoractomy / VATS)	1.389	0.187	55.351	0.000*	4.010	2.781∼5.781
Type of surgical resection (wedge resection / segmentectomy / lobectomy / pneumonectomy / others)	0.125	0.083	2.243	0.134	1.133	0.962∼1.334
Neoadjuvant chemotherapy (no /yes)	-0.379	0.224	2.877	0.090	0.684	0.442∼1.061

* Statistically significant

**Table 3:** Comparison of length of stay and cost between the two groups [M(Q1, Q3)] (*n* = 1317)

Group	Total number	Length of ICU admission (day)	Average days of hospitalization (day)	Average costs of hospitalization (yuan)
Abdominal distension group	182	1(1,1)	9(7,10)	37852.55(26936.33,50992.23)
Non-distended group	1135	1(1,1)^#^	7(5,9) ^#^	32182.73(25335.28,42039.49)
*P*		0.333	0.000	0.032

#: 2 dead

**Table 4:** Comparison of complication rates between the two groups [n(%)] (*n* = 1317)

Complications	Total number	Lung infection	Cardiac arrhythmia	Hemorrhage
Abdominal distension group	182	18(9.89)	26(14.29)	5(2.75)
Non-distended group	1135	89(7.84)	134(11.81)	17(1.50)
*P*		0.348	0.342	0.222

**Table 5:** Comparison of length of stay and cost between the two patients with different complications and types of surgical resection [M(Q1, Q3)] (*n* = 1317)

Complications	Total number	Average days of hospitalization (day)	Average costs of hospitalization (yuan)
Lung infection			
no	1252	7(5,9)	31603.51(25766.13,42115.36)
yes	65	9(8,11) ^#^	39862.16(35475.19,53179.13)
*P*		0.000*	0.000*
Cardiac arrhythmia			
no	1157	8(6,9)	34072.57(25317.28,41793.43)
yes	160	8(7,9)	37152.64(27429.26,43174.57)
*p*		0.412	0.179
Hemorrhage			
no	1295	8(6,9)	33918.50(24179.31,41546.17)
yes	22	8(7,10)	36879.14(26147.35,45339.50)
*p*		0.378	0. 183
Type of surgical resection			
Wedge resection	155	5(4,7)	29594.24(25225.56,38082.00)
Segmentectomy	329	7(5,10)	31127.87(25017.46,40320.94)
Lobectomy	724	7(5,9)	33971.79(26376.10,44204.08)
Pneumonectomy	13	9(8,11)	35878.20(27222.60,60434.11)
Others	96	8(7,11)	34792.56(22022.33,41638.51)
*P*		0.000*	0.001*

* Statistically significant

## Discussion

The results of the current study showed that the incidence of abdominal distension in postoperative lung cancer patients in ICU was 13.8%, which is close to the incidence of cardiopulmonary complications (18.4%) [9]. The results of multifactorial logistic regression analysis showed that women, older and patients with open thoracotomy took a higher risk of experiencing abdominal distention, which were independent risk factors for postoperative abdominal distention in lung cancer.

Abdominal distension is a subjective sensation of abdominal swelling and accompanied by visible increase in abdominal girth, which can occur in any part of the abdomen (upper, middle, lower or whole abdomen) and has a complex etiology and pathogenesis that is usually the result of multiple factors which are not fully understood [13, 14]. Abdominal distension may often lead to delayed feeding, anxiety and sleep disturbance, which may impair patients’ recovery [15]. Previous studies have shown that abdominal distention may be associated with pathophysiological factors such as altered microbiota, abnormal gastrointestinal dynamics, abdominophrenic dyssynergia (APD) and visceral hypersensitivity reactions [11]. It has also been shown that abdominal distension may be associated with operative stress, postural immobilization, and slowed gastrointestinal motility due to perioperative use of opioids and anesthetics [12].

The study results showed that the incidence of abdominal distension was significantly higher in women (19%) than in men (13.11%) (*P* < 0.01), similar to the findings of Jiang [16]. The study of Xu [17] suggested that women are relatively slow to recover from postoperative gastrointestinal tract, and women are more sensitive than men in terms of emotional performance and more prone to negative emotions such as anxiety and depression [18]. As reported in the literature [19], the prevalence of clinically anxiety and depression in female lung cancer patients was as high as 30% and 24.7%, respectively, and female cancer patients were almost two times more likely than males (24.0% versus 12.9%)to report clinical levels of anxiety, which to some extent aggravate the incidence of abdominal distension. In addition, influenced by traditional culture, male patients in China show a higher tolerance to their symptoms and have a relatively low reporting rate of abdominal distension [17]. Therefore, the above factors may lead to a higher incidence of abdominal distension in women than men.

This study showed that the risk of abdominal distension in postoperative lung cancer patients increases with age (OR = 1.154, 95%CI = 1.022∼1.304). It is possible that when age increases, people’s physical function declines, metabolism slows down, and gastrointestinal peristalsis slows down, which can easily lead to gastric retention and abdominal distension.

The incidence of abdominal distension was significantly higher in patients undergoing open thoracotomy (21.44%) than in patients undergoing thoracoscopic surgery (6.94%) (*P* < 0.01). With the advancement of surgical techniques, video-assisted thoracoscopic surgery (VATS) is gradually used as an alternative to open thoracotomy, which has a smaller incision and fewer postoperative complications compared with traditional open thoracotomy, and is more conducive to reducing postoperative pain and inflammatory immune response in patients and promoting rapid postoperative recovery, thereby shortening hospital stay, improving patients’ quality of life, and even improving long-term survival [20–25].

The increase of hospitalization days and hospitalization costs may be the result of multiple factors, including abdominal distension, postoperative complications (lung infection), and the type of surgical resection (pneumonectomy). The results of the current study showed that the number of days and the cost of hospitalization for patients with abdominal distension and postoperative complications were higher than normal group (*P*＜0.01), similar to the findings of Schulze [15] and Li [26]. It indicates that postoperative abdominal distention and lung infection may prolong patients’ hospital stay, and increase medical costs [27].

Recent advances in early detection and screening of lung cancer decrease the number of patients with advanced diseases [28, 29]. However, pneumonectomy is still necessary for 10% of patients undergoing surgical resection [30]. Pneumonectomy greatly increases the risk of postoperative complications and mortality [31, 32]. The results of the current study showed that the length of ICU admission was higher in patients with pneumonectomy (approximately 1.69 days) than in patients with other types of surgical resection (approximately 1.16 days), in addition, patients with pneumonectomy combined with a higher incidence of abdominal distension, which further contributed to higher hospitalization costs and days than other types of surgical resection.

Therefore, medical staff should fully evaluate female patients before surgery and understand the psychological changes of patients at different ages and providing them with targeted health education, reasonably choose the surgical method, and strengthen the understanding, preventing and managing of perioperative abdominal distension in lung cancer patients, which is important to prevent the occurrence of postoperative abdominal distension in lung cancer and reduce the length of hospitalization and economic burden of patients.

The study had several limitations. First, as a single-center retrospective cohort study, it included data obtained from secondary data sources (the HIS system). Consequently, information bias could be present. Second, the study was conducted at a single hospital, and therefore it is not representative of the whole region or country. Third, only relevant influencing factors were collected and analyzed, and the inclusion of indicators may not be comprehensive. Subsequent prospective, multicenter, and large-sample studies should be conducted.

## Conclusions

In conclusion, the incidence of abdominal distension was high in postoperative lung cancer patients in ICU, which is closely related to gender, age and surgical approach, and may extend the patients’ hospital stay and increase medical costs.

## Data Availability

The datasets analyzed during the current study are available from the corresponding author on reasonable request.

## Abbreviations

ICU: Intensive Care Unit
HIS system: Hospital Information System
BMI: Body Mass Index
VATS: Video-Assisted Thoracoscope
